# Insecticidal Constituents and Activity of Alkaloids from *Cynanchum mongolicum*

**DOI:** 10.3390/molecules200917483

**Published:** 2015-09-21

**Authors:** Yang Ge, Pingping Liu, Rui Yang, Liu Zhang, Hongxing Chen, Ibrahima Camara, Yiqing Liu, Wangpeng Shi

**Affiliations:** 1 College of Agronomy and Biotechnology, China Agricultural University, Beijing 100193, China; E-Mails: 13120154491@163.com (Y.G.); liudoubleping@163.com (P.L.); 18010128390@163.com (R.Y.); zhangliucau@163.com (L.Z.); 15600660156@163.com (H.C.); kamibrahimsidik41@gmail.com (I.C.); 2 Collaborative Innovation Center of Special Plant Industry, Chongqing University of Arts and Sciences, Chongqing 402160, China; E-Mail: liung906@163.com

**Keywords:** *Cynanchum mongolicum*, alkaloids, insecticidal constituents, insecticidal activity, *Spodoptera litura*, *Lipaphis erysimi*

## Abstract

Based on MS and NMR data and bioassay-guided tracing, three insecticidal alkaloids **I**, **II** and **III** from *Cynanchum mongolicum* were identified to be antofine *N*-oxide, antofine and tylophorine. Alkaloid **I** was more toxic than alkaloids **II** and **III**, but they were less active against *Spodoptera litura* than total alkaloids. The contact toxicity from these alkaloids against the aphid *Lipaphis erysimi* was significant, as the 24 h-LC_50_ values of alkaloids **I**, **II**, **III** and total alkaloids were 292.48, 367.21, 487.791 and 163.52 mg/L, respectively. The development disruption of *S. litura* larvae was tested, the pupation and emergence rates of *S. litura* decreased and the acute mortality of *S. litura* increased significantly by day 3 after being injected in their body cavity with 10–40 mg/L of total alkaloid. The ecdysone titer of treated *S. litura* larvae and prepupae declined with increasing alkaloid concentration. The alkaloids of *Cynanchum mongolicum* are potential insect growth inhibitors.

## 1. Introduction

*Cynanchum mongolicum* AL. Iljinski (Asclepiadaceae) is an herb found in grasslands in several parts of the world, including northern China, and has been used to control crop pests. More than 200 compounds have been isolated from *C. mongolicum*, including dozens of alkaloids. The insecticidal activity of mixtures of these alkaloids has been tested against a range of infamous insect pests such as *Spodoptera litura* Fabricius and *Lipaphis erysimi* (Kaltenbach) [1] which are able to develop continuously throughout the year in greenhouses, and in many crops these pests can reach high population levels, causing substantial economic losses [2]. The insecticidal constituents, activities and the exact physiological mechanism causing the insecticidal activity of these alkaloids have not been determined. Because of these species’ potential to damage crops, pesticides have been used extensively against them. As a result of the intensive selection pressure, *S. litura* and *L. erysimi* now show high levels of resistance to many commonly used chemical insecticides [3]. In contrast, extracts of *C. mongolicum* provide good control of *S. litura* and *L. erysimi*, and the general nature of the insecticidal activity (molting disruption) of *C. mongolicum* extractions is known [1]. There is no information on the insecticidal activities of monomeric alkaloids from *C. mongolicum*. Here, the details of the insecticidal constituents, activities, action modes and physiological mechanism against insects have been researched. The effects of *C. mongolicum* alkaloids on molting hormone (MH) titers, and the resultant growth and development of *S. litura* larvae were investigated.

## 2. Results

### 2.1. Alkaloid Identification

**Figure 1:**
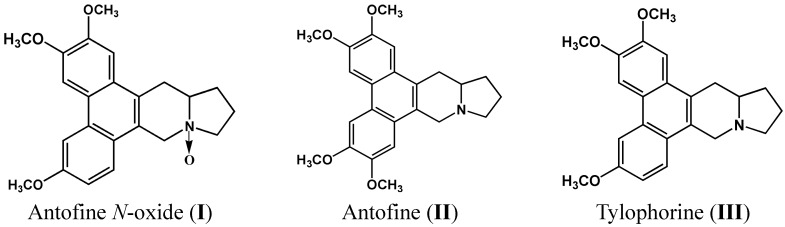
Structures of bioactive compounds isolated from *Cynanchum mongolicum*.

Three bioactive compounds were isolated from *C. mongolicum.* The compounds were isolated using bioassay-guided fractionation and then identified based on their spectroscopic data in comparison with literature values as antofine *N*-oxide (**I**), antofine (**II**) and tylophorine (**III**) (Figure 1).

### 2.2. Acute Lethal Toxicity of Different Alkaloids against Wingless Adult Lipaphis erysimi Aphids

The alkaloids of *C. mongolicum* (Maxim) were very toxic to wingless adult aphids *L. erysimi* (Table 1). Mortality was highest when aphids were exposed to all three alkaloids combined OR to the total alkaloid fraction. The mortalities of aphids reached a peak at 24 h after treatment. The regression analysis between concentration and mortality showed those correlation coefficients are above 0.90. With 1 day exposure, the respective LC_50_ values of alkaloids **I**, **II**, **III**, total alkaloids and imidacloprid were 292.48 mg/L, 367.21 mg /L, 487.79 mg/L, 163.52 mg/L and 8.087 mg/L, respectively.

**Table 1:** Toxicity of *C. mongolicum* alkaloids against wingless adult aphids *Lipaphis erysimi*.

Alkaloids	Slope (SE)	LC_50_ (mg/L)	95% Confidence Interval (mg/L)	Chi-Square Value	Correlation Coefficient
**I**	5.22 (0.93)	292.48	281.32~313.83	12.54	0.96
**II**	7.67 (0.31)	367.21	307.21~421.30	14.37	0.92
**III**	8.26 (0.71)	487.79	439.11~538.91	12.51	0.98
Total alkaloids	3.86 (0.17)	163.52	102.66~231.52	8.93	0.96
Imidacloprid	0.37 (0.12)	8.087	0~33.51	4.16	0.89

### 2.3. Development Disruption

**Figure 2:**
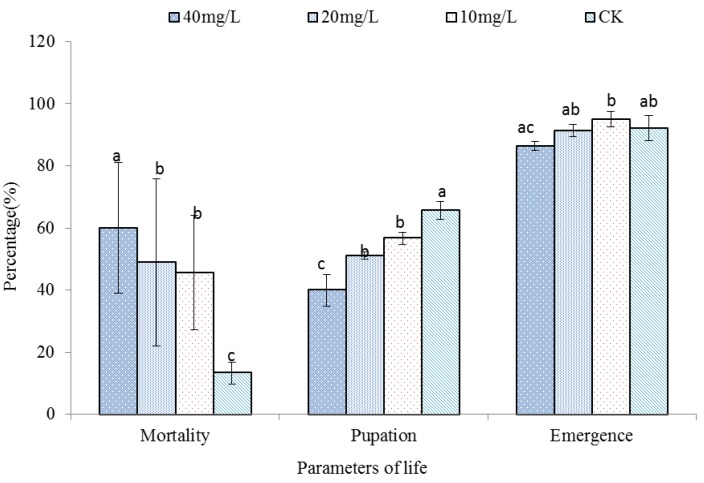
Effect of total alkaloids on mortality, pupation and emergence of *Spodoptera litura*. Bar (±SE) followed by the same letter within a parameter are not significantly different at *p* < 0.05 based on Duncan’s multiple range tests for pupation, emergence, and mortality. CK: 70% ethanol.

By the third day following treatment with 10, 20 or 40 mg/L total alkaloids from *C. mongolicum*, the mortality of *S. litura* was significantly (*p* < 0.05) higher than controls, being 45% for the 10 mg/L treatments, 51% for the 20 mg/L treatments and 60% for the 40 mg/L treatments (Figure 2).

Of the *S. litura* that survived, there was a further effect on reduced pupation ranging from an 8.9% reduction at the lowest dose to 25.6% at the highest dose of total alkaloids. Of those that did pupate, there was a further minor effect of a 0%–5.8% reduced emergence from the pupal stage (Figure 2).

### 2.4. Molting Hormone Titers of Treated Larvae or Prepuae

**Figure 3:**
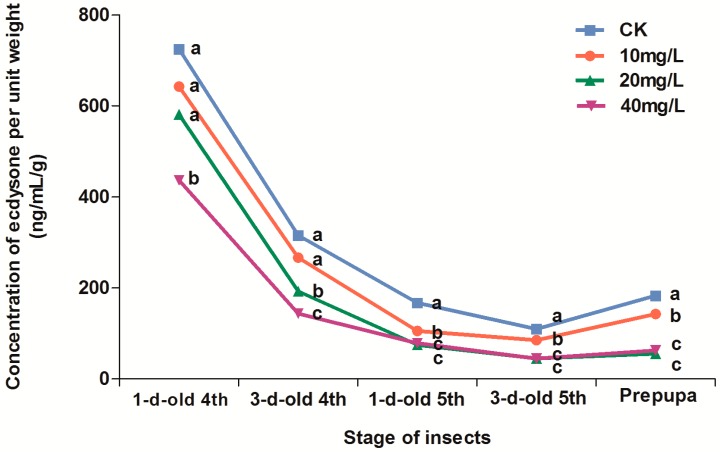
The concentration of ecdysone per unit weight of *Spodoptera litura* larvae or prepupae after treatment with different concentrations of total alkaloids. Bar (± SE) (4 replicates) followed by the same letter within a stage are not significantly different at *p* < 0.05 using Duncan’s multiple range test. CK: 70% ethanol.

Titers of molting hormone decreased with increased concentration of total alkaloids (from 0–40 mg/L). Titers of molting hormone were highest for 1-day-old 4th instar larvae. For 1-day-old 4th instar larvae, molting hormone titers in *S. litura* larvae treated with the alkaloid at 40 mg/L decreased significantly compared to the other treatments. For 3-days-old 4th instar larva, molting hormone titers in *S. litura* larvae treated with alkaloids at both the 20 mg/L and 40 mg/L were significantly lower than that at 0–10 mg/L. For 5th instar larvae at 1 and 3 days old, and for prepupae, molting hormone titers in *S. litura* larvae treated with alkaloids at 20–40 mg/L decreased significantly, though there was also a slight, but significant, decrease at the 10 mg/L treatments (Figure 3).

### 2.5. Standard Curve of 20-Hydroxyecdysone.

Analyses of known concentrations over the range of 0.16 to 100 ng of 20-hydroxyecdysone found that the peak area predicted concentration as Y = 60.234X + 24.184, where X is molting hormone concentration and Y is the area under the HPLC, with R^2^ = 0.9999.

## 3. Discussion

Here three insecticidal ingredients (alkaloids **I**, **II** and **III**) were isolated from the total alkaloid complex of *Cynanchum mongolicum* by bioassay-guided fractionation and their structures were identified to be antofine *N*-oxide, antofine and tylophorine, repectively. These three alkaloids have been isolated before from this species and it was already known that these alkaloids showed important antineoplastic and antiviral properties [4]. However, little was known about the insecticidal activity of antofine *N-*oxide and tylophorine. The research results indicated that these three alkaloids exhibited marked insecticidal activity on *S. litura* and *L. erysimi*. Alkaloid **I**, is the main insecticidally active component because it was found in the highest concentration and has the lowest LD_50_, and while alkaloids **II** and **III** are of lesser importance individually, they no doubt contribute to the higher toxicity seen in the total alkaloids. The higher toxicity of the total alkaloid mixture occurred not only because there are likely to be other components that were not isolated that might potentiate the effects of the alkaloids, but also because of possible synergistic effects between the alkaloids that increase their overall toxicity [5]. While the activity of the extracts on *L. erysimi* is lower than that of imidacloprid, the insecticidal activity of these alkaloids from *C. mongolicum* was significantly stronger than that of the extracts from *Aloe barbadensis*, *Carica papaya* and *Xanthium sibirium* [6].

In the study, the insecticidal physiological mechanisms of alkaloids from *C. mongolicum* were investigated. These results show the effects of alkaloids from *C. mongolicum* on molting hormone titers, growth, and development of *S. litura*. Higher alkaloid concentrations resulted in greater developmental disruption and mortality, especially by 72 h post-treatment. The alkaloids from *C. mongolicum* appear to disrupt insect hormone balances in a similar manner to that shown for azadirachtin. Previous reports found that treatment with 800 mg/L of the extracts led to more than half of the resulting *S. litura* pupae not molting into adults. Also, the developmental time (from third instar to emergence) of insects treated with alkaloids was increased [7].

Molting of insects is a complicated physiological reaction affected by several hormones (prothoracicotropic hormone, molting hormone, and juvenile hormone). The secretion of prothoracicotropic hormone, PTTH, promotes the production of molting hormone, which is integral for insects’ transition to adulthood. But juvenile hormone, JH, represses the synthesis and secretion of molting hormone [8]. If the synthesis or secretion of these hormones is disrupted, the metamorphosis and development of insects is compromised [8]. In this study, *S. litura* had two secretion peaks of molting hormone between the 4th instar and pupation, but the two peaks were both disrupted and weakened after treatment with total alkaloids (Figure 3). In this study we tested the action of the total alkaloids, the mechanism of the individual active ingredients needs to be analyzed further. Plant-derived insect growth regulators appear mainly to interfere with the endocrine system or chitin synthesis [9]. The disruption of the endocrine system and hormone balance of insects by azadirachtin has been clearly demonstrated in previous studies [10]. The alkaloids from *C. mongolicum* appear to disrupt insect hormone balances in a similar manner to that shown for azadirachtin, which may also compete to bind to specific membrane proteins [10]. Further work is needed on the effects of the alkaloids on the endocrine glands of insects using morphological anatomy and isotope labeling, to identify the exact mechanism of alkaloid action.

## 4. Experimental Section

### 4.1. General Information

^1^H-NMR and ^13^C-NMR spectra were obtained on ACF300 (300 MHz (^1^H)) and AMX500 (500 MHz (^1^H)) spectrometers (Bruker, Germany) using deuterochloroform (CDCl_3_) as the solvent with tetramethylsilane (TMS) as the internal standard, and electron impact ionone mass spectra were determined on a Micromass VG7035 mass spectrometer (VG Micromass, Manchester, UK) at 70 eV. Silica GF254 (10–40 μm) for PTLC and silica gel (200–300 mesh) for column chromatography were purchased from Qingdao Marine Chemical Company (Qingdao, China). All other chemicals were of reagent grade.

### 4.2. Plant Materials

Samples of the herb *Cynanchum mongolicum* were collected from the Inner Mongolia Erdos prairie in September 2006. The herb was identified by Prof. Qingru Wu (Department of Biology, Inner Mongolia Polytechnic University, Huhehaote, China), and a voucher specimen (NO: 96007) was lodged in the laboratory of the Inner Mongolia Polytechnic University.

### 4.3. Insects

The *S. litura* larvae used in the study came from a laboratory colony originating from stock obtained from the Laboratory for Biological Control of the China Agricultural University in Beijing, which was established with *S. litura* collected during 2003 in Yaan, Sichuan, China. The larvae were reared individually in glass tubes (13 × 100 mm) at 27 ± 1 °C, a light-dark cycle of 14:10 h, and 60%–70% RH. Larvae were fed an artificial diet*,* and feces were removed daily [11]. All assays were conducted under these same conditions. The third-instar larvae were selected for testing. The aphids *Lipaphis erysimi* came from a laboratory colony originating from stock obtained from the Laboratory for Biological Control of the China Agricultural University in Beijing, and were reared in turnip in pot at 28 ± 2 °C, a light-dark cycle of 10:14 h, and 30%–50% RH. Wingless adults were selected for testing.

### 4.4. Alkaloid Extraction, Preparation and Identification

*Cynanchum mongolicum* medicinal plants were cleaned and dried in an outdoor area under full shade. Dried plants were finely ground (HY-04B Beijing Xinhuanya Technology Ltd., Beijing, China) and sieved through a 40-mesh sieve, and the resulting powder kept in a sealed container until use.

The alkaloids were extracted following the methods outlined in Chen *et al.* [12]. Twenty kg of powdered dried plant material was soaked in industrial alcohol (95.6%, Beijing Chemical Reagent Ltd., (B.C.R.L.) Beijing, China) containing 0.1% hydrochloric acid (37%, B.C.R.L.) for 72 h. The solution was then extracted through filter paper (20–30 µm, B.C.R.L.) using a vacuum pump (SHB-III; Zhengzhou Great Wall Industrial and Trading Co., Zhengzhou, China). The extracted solution was then dissolved in 2% hydrochloric acid solution, and insoluble particles removed through filtration and extraction with chloroform (RD1.4832 [[20/4 °C]). The solution’s pH was then adjusted to a range of 9–11 with sodium hydroxide (98%, B.C.R.L.). Potassium bismuth iodide or I2-KI solution was then added to the alkaloid-containing solution to produce a yellow-to-orange or brown precipitate, demonstrating that the solution contained alkaloids [13]. The solution was then subjected to chloroform extraction and vacuum concentration five times with a rotary evaporator (RE52-98, Shanghai Yarong Biochemical Instrument Factory, Shanghai, China) to isolate the desired alkaloids. The crude alkaloid extract (40 g) was subjected to column chromatography on a silica gel column and eluted with a step wise gradient of CH_2_Cl_2_/CH_3_OH (25:1, 15:1, 5:1, 5:2 by volume) [14], 0.51 g total alkaloid was prepared for these experiments. The insecticidal fraction A_1_ was further separated on silica gel column with elution of CH_2_Cl_2_/CH_3_OH (12:1 by volume) to give alkaloid I (0.11 g). The insecticidal fraction A_2_ was further purified using silica gel column with CH_2_Cl_2_/CH_3_OH (9:1 by volume) to yield alkaloid II (0.06 g). The insecticidal fraction A_3_ was further purified using a silica gel column with CH_2_Cl_2_/CH_3_OH (6:1 by volume) to yield alkaloid **III** (0.02 g).

*Alkaloid* **I**: colorless needle-like crystals, m.p. 197~198 °C (CH_3_OH); ${\left[\alpha \right]}_{D}^{25}$: 65° (CHCl_3_), MS (70 eV) *m*/*z* (%): 379 (M^+^, 3), 363 (25), 294 (17). The spectroscopic data is consistent with that listed in the literature [15], so alkaloid I was identified as antofine *N*-oxide. The chemical structure is given in Figure 1 (**I**); ^13^C- and ^1^H-NMR spectra of antofine *N*-oxide: Figures S1 and Figures S2.

*Alkaloid* **II**: light yellow needle-like crystals, m.p. 210~212 °C; ${\left[\alpha \right]}_{D}^{25}$: 195° (CHCl_3_), MS (70 eV) *m*/*z* (%):364 (M^+^, 79), 294 (100), 279 (17). The spectroscopic data is consistent with that listed in the literature [16], so alkaloid **I** was identified as antofine. The chemical structure is given in Figure 1 (**II**); ^13^C**-** and ^1^H-NMR spectra of antofine: Figures S3 and Figures S4.

*Alkaloid* **III**: light yellow needle-likes crystals, m.p. 210~212 °C (CH_3_OH); ${\left[\alpha \right]}_{D}^{25}$: 95° (CHCl_3_), MS (70 eV) *m*/*z* (%): 393 (M^+^, 35), 324 (100), 294 (17). The spectroscopic data is consistent with that listed in the literature [17], so alkaloid I was identified as tylophorine. The chemical structure is given in Figure 1 (**III**); ^13^C- and ^1^H-NMR spectra of tylophorine: Figures S5 and Figures S6.

### 4.5. Acute Lethal Toxicity Test

The acute lethal toxicity of the alkaloids was studied with the leaf dipping method. Wingless adults of the aphid *Lipaphis erysimi* were selected for test. The wax on the surface of bean leaf was scraped off with knife, and then a 2 × 3 cm^−1^ rectangle was punched out of the leaf. The leaves were then dipped in the selected solutions for five seconds, and then air-dried. The alkaloids **I**, **II**, **III** and total alkaloids were suspended in alcohol and the following concentrations were used: 0 (Control, 70% ethanol only), 50, 100, 200, 400, 800 mg/L. 96% imidacloprid was used as positive control, because 70% ethanol poses no significant risk or alteration of growth patterns to the insect, it is a suitable negative control [7]. Thirty wingless adults were put on the treated or control leaves placed in a glass Petri dishes (Φ = 9 cm). Each treatment was repeated three times. All subsequent incubation conditions were as those described above. Mortality was recorded after 24 h following consumption of the leaf disc. A toxic regression equation was calculated and an LC_50_ obtained using probit analysis.

### 4.6. Development Disruption Tests

Third instar *S. litura* larvae from our laboratory colony were injected in their body cavity with 1 μL/individual of either a 10, 20, or 40 mg/L solution of total alkaloids using a micro-injector. Control larvae were injected with 1 μL/individual of solvent (70% ethanol) (CK). Each treatment group contained 30 larvae and each treatment was repeated three times. Larvae were then reared individually as described above until they became adults or died. Acute mortality was measured before pupation and emergence, and then the pupation and emergence of surviving individuals were recorded until they became adults.

### 4.7. Estimation of Molting Hormone Titers

Hemolymph for the estimation of molting hormone titers in treated or control *S. litura* larvae was collected from 1 and 3 day old 4th instar larvae, 1 and 3 day old 5th instar larvae and prepupae. Each treatment contained 50 insects and was replicated four times. Each insect was weighed with an electronic balance (TP 214, Denver Instrument Co. Denver, CO, USA). The extraction and quantification methods used followed Wilson and Gryan [18]. Hemolymph was extracted from larvae by cutting off the tip of a proleg and inserting a capillary tube. For prepupae, an incision was made in the dorsum and a capillary tube inserted to collect hemolymph. After collection, each hemolymph sample was placed in 2 mL of 75% cold methanol and stored in a freezer at −20 °C until analyzed. For analysis, solutions were shaken and then centrifuged at 10000 r/min for 10 min at 4 °C. The supernatant fluid was then evaporated to dryness with N_2_ gas. The dry product was then added to 10 mL of water and an equal volume of chloroform. This mixture was then shaken and centrifuged a second time at 10,000 r/min for 10 min at 4 °C the supernatant fluid was collected, the sediment was then re-extracted with pure water as above, and the new supernatant fluid collected and added to again., The collected supernatant fluids were then combined and passed through a filter needle, which was eluted with 3.5 mL of 25% methanol, and then eluted again with 3.5 mL of 80% methanol. The final solution was evaporated with N_2_ and pure methanol was added to create a constant volume 10 mL for HPLC analysis.

Titers of molting hormone were measured using an Agilent 1100 (Santa Clara ,CA, USA), equipped with an Agilent eclipse XDB-C18 column (4.6 mm, ID × 150 mm), using a methanol–water mixture (55:45) as the solvent, a flow velocity of 1 mL/min, and a column temperature of 25 °C. The volume of the injected samples was 20 μL. Detection of molting hormone was based on peaks at ultraviolet wavelengths of λ = 250 nm. Final quantification was based on the area under the chromatographic peak for molting hormone, with reference to a standard curve of 20-hydroxyecdysone. 

### 4.8. Standard Curve of 20-Hydroxyecdysone Concentration

A series of 20-hydroxyecdysone samples of different known concentrations (0.16, 0.8, 4, 20, and 100 ng/μL) were prepared and the samples were analyzed with HPLC under the conditions described above. The linear regression equation between areas under chromatographic peaks and these known concentrations was used to quantify 20-hydroxyecdysone concentrations from experimental samples using their chromatographic peak areas.

### 4.9. Statistical Analyses

Initially the homogeneity of variances was determined by the Duncan’s multiple range tests for pupation, emergence, and mortality. Thereafter one-way analysis of variance was applied to detect the significant differences (*p* < 0.05) between samples. The analysis was performed with SPSS 17 statistics package (IBM, SPSS, Armonk, NY, USA).

## 5. Conclusions

Three insecticidal alkaloids **I**, **II** and **III** from *C. mongolicum* were identified to be antofine N-oxide, antofine and tylophorine, and alkaloid **I** was more toxic than alkaloids **II** and **III**. The contact toxicity from these alkaloids against the aphid *L. erysimi* was significant, but these alkaloids only showed the stomach toxicity against *S. litura* and inhibited the development of *S. litura*.

## Supplementary Materials

Supplementary materials can be accessed at: http://www.mdpi.com/1420-3049/20/09/17483/s1.
