# Supplementary material for: Insecticidal Constituents and Activity of Alkaloids from Cynanchum mongolicum
Source: Molecules. 2015 Sep 21;20(9):17483–92. doi: 10.3390/molecules200917483 (PMC6331922; doi:10.3390/molecules200917483)
Supplement: Supplementary file 1 [file molecules-20-17483-s001.pdf]

Supplementary Information

Content

Figure S1. ^{13}C-NMR spectra of *N*-oxide antofine S2

Figure S2. ^{1}H-NMR spectra of *N*-oxide antofine S3

Figure S3. ^{13}C-NMR spectra of antofine S4

Figure S4. ^{1H}-NMR spectra of antofine S5

Figure S5. ^{13}C-NMR spectra of tylophorine S6

Figure S6. ^{1}H-NMR spectra of tylophorine S7

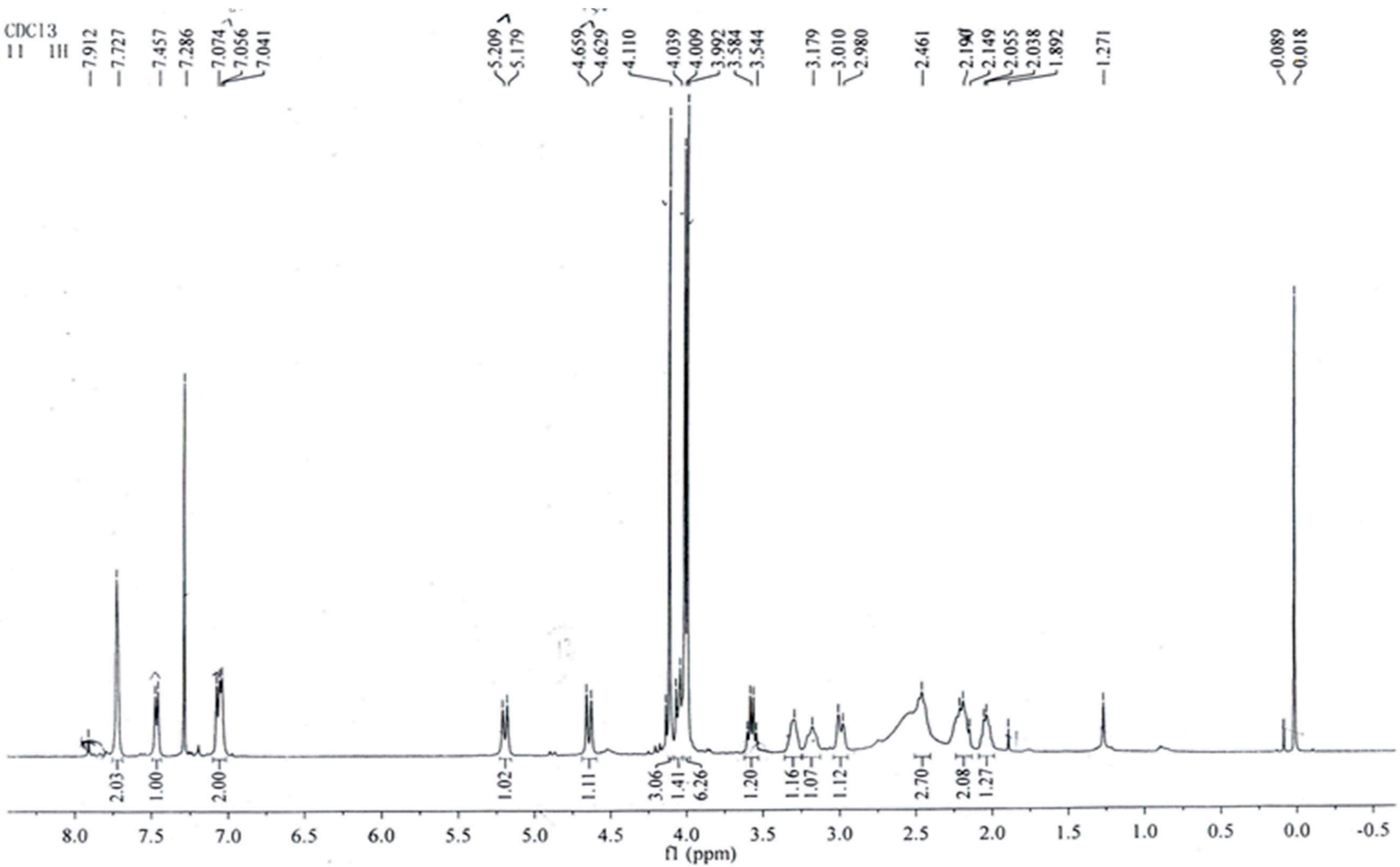

Figure S1. ^{13}C-NMR spectra of *N*-oxide antofine.

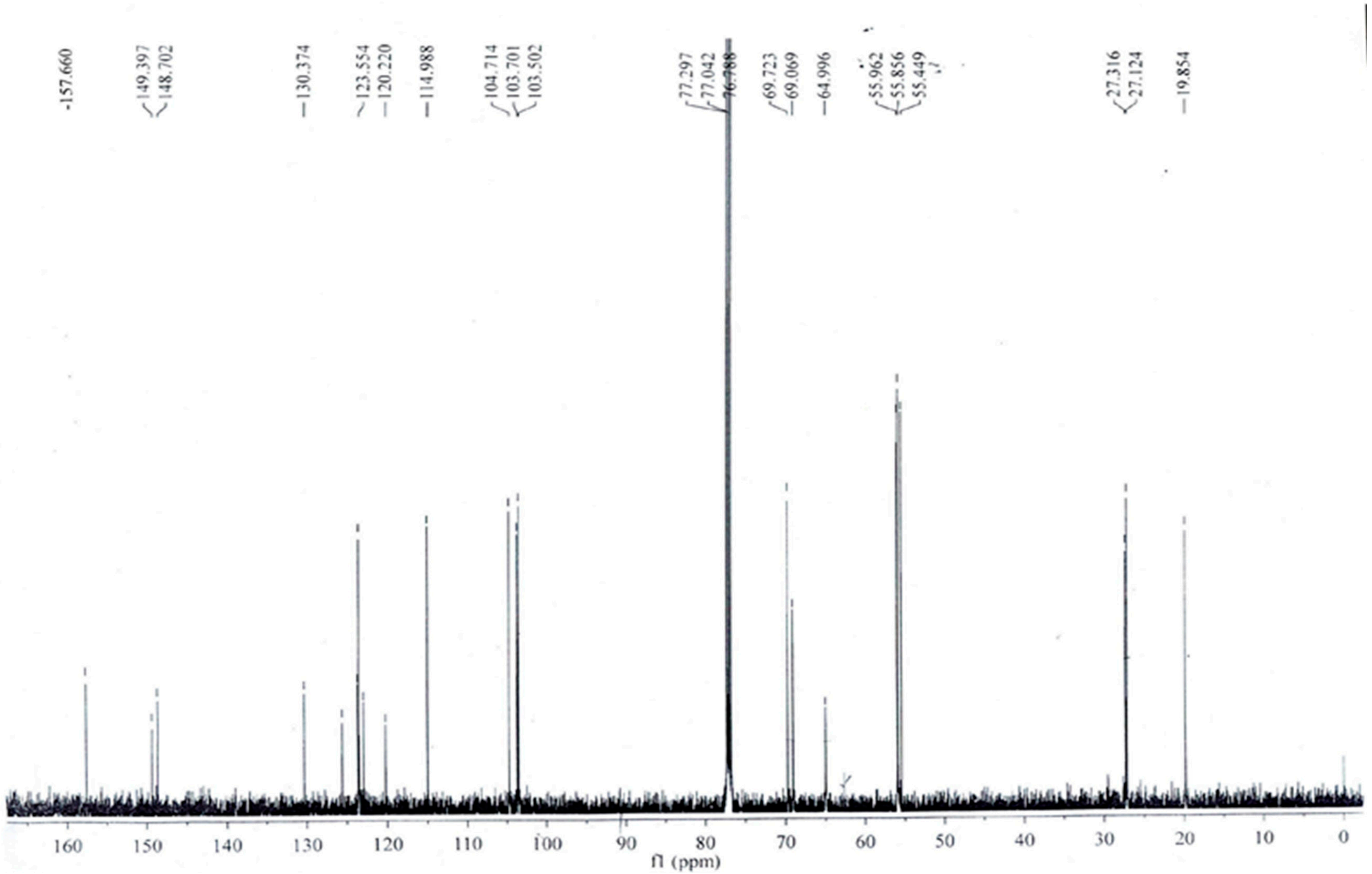

Figure S2. ^{1}H-NMR spectra of *N*-oxide antofine.

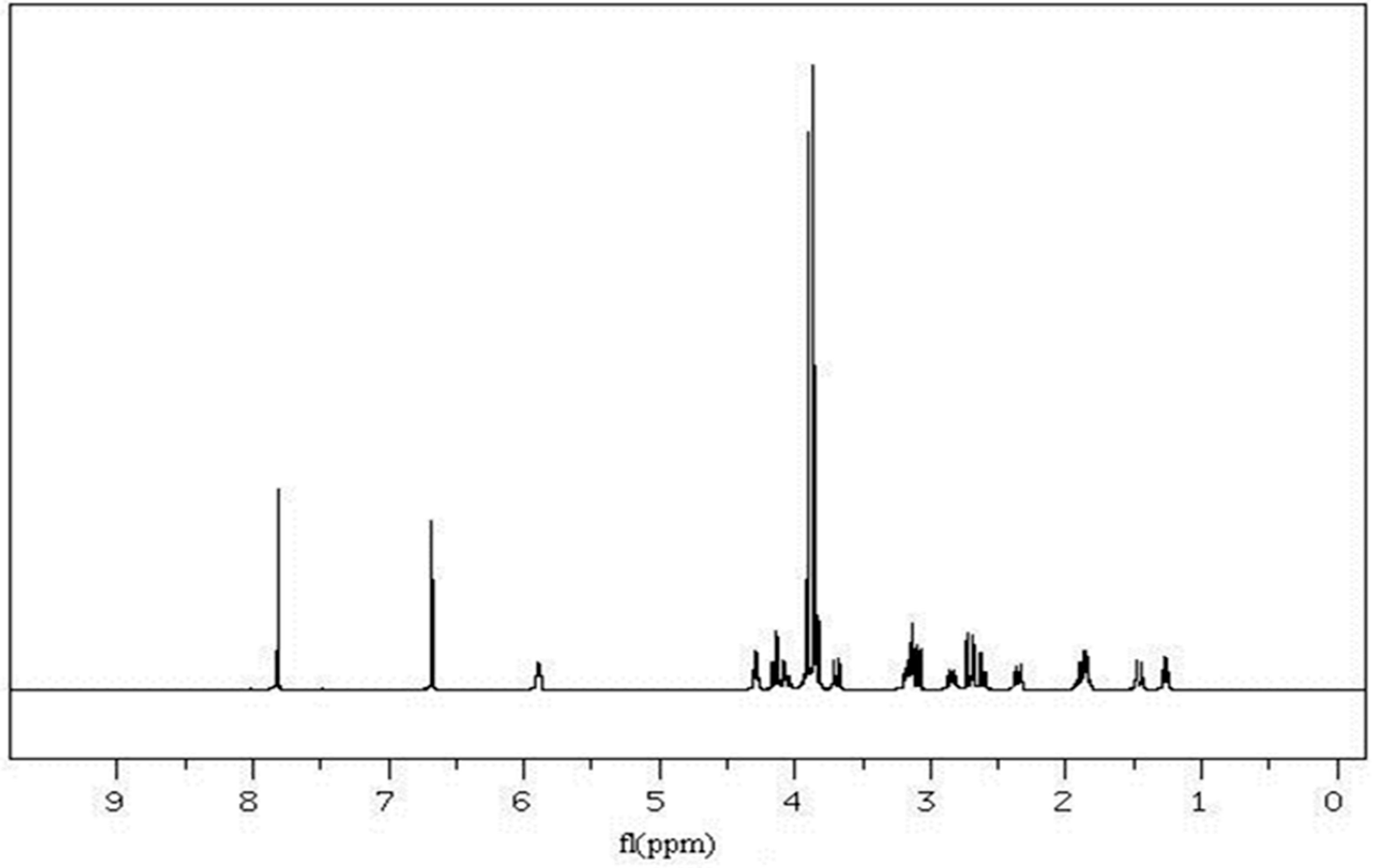

Figure S3. ^{13}C-NMR spectra of antofine.

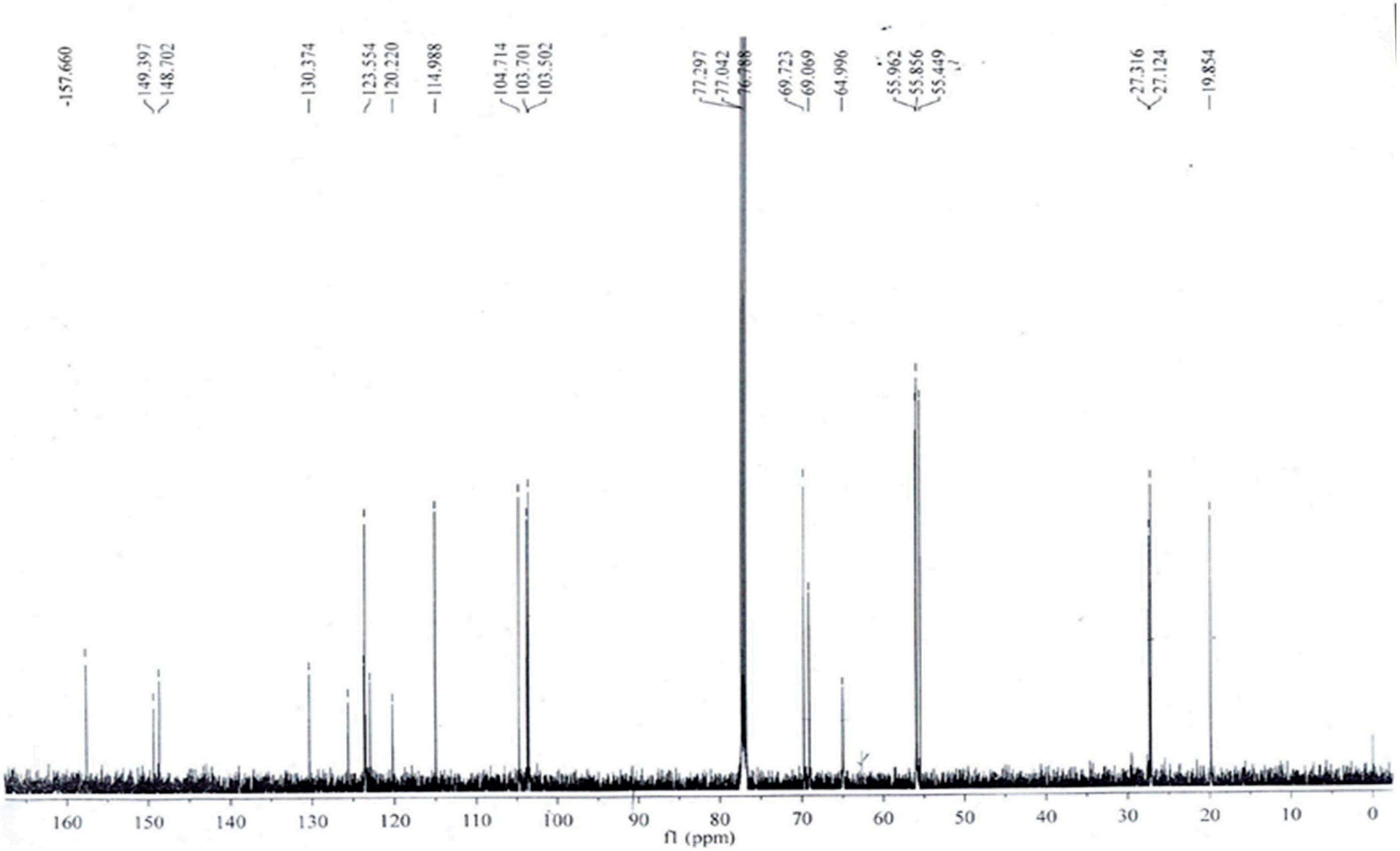

Figure S4. ^{1}H-NMR spectra of antofine.

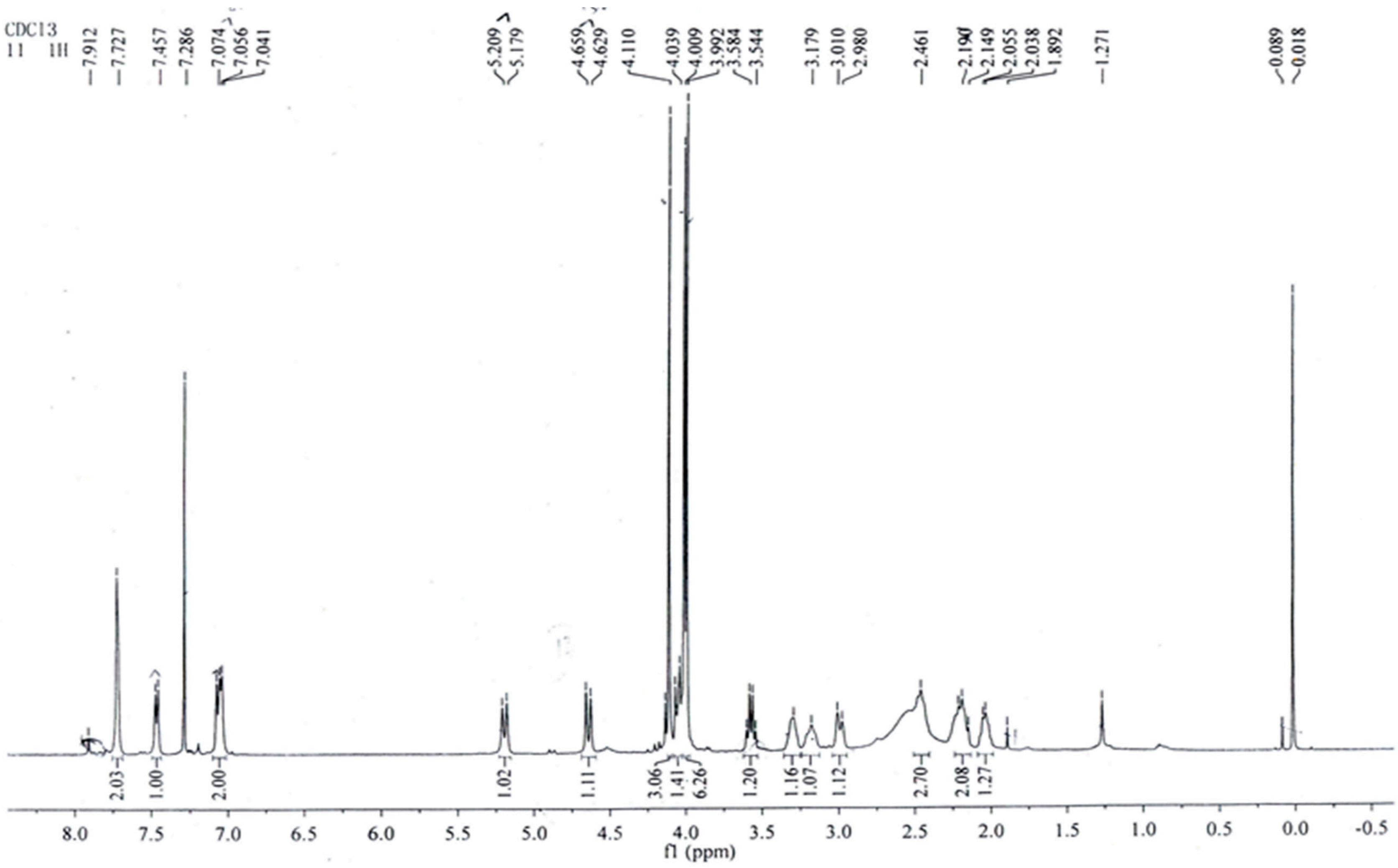

Figure S5. ^{13}C-NMR spectra of tylophorine.

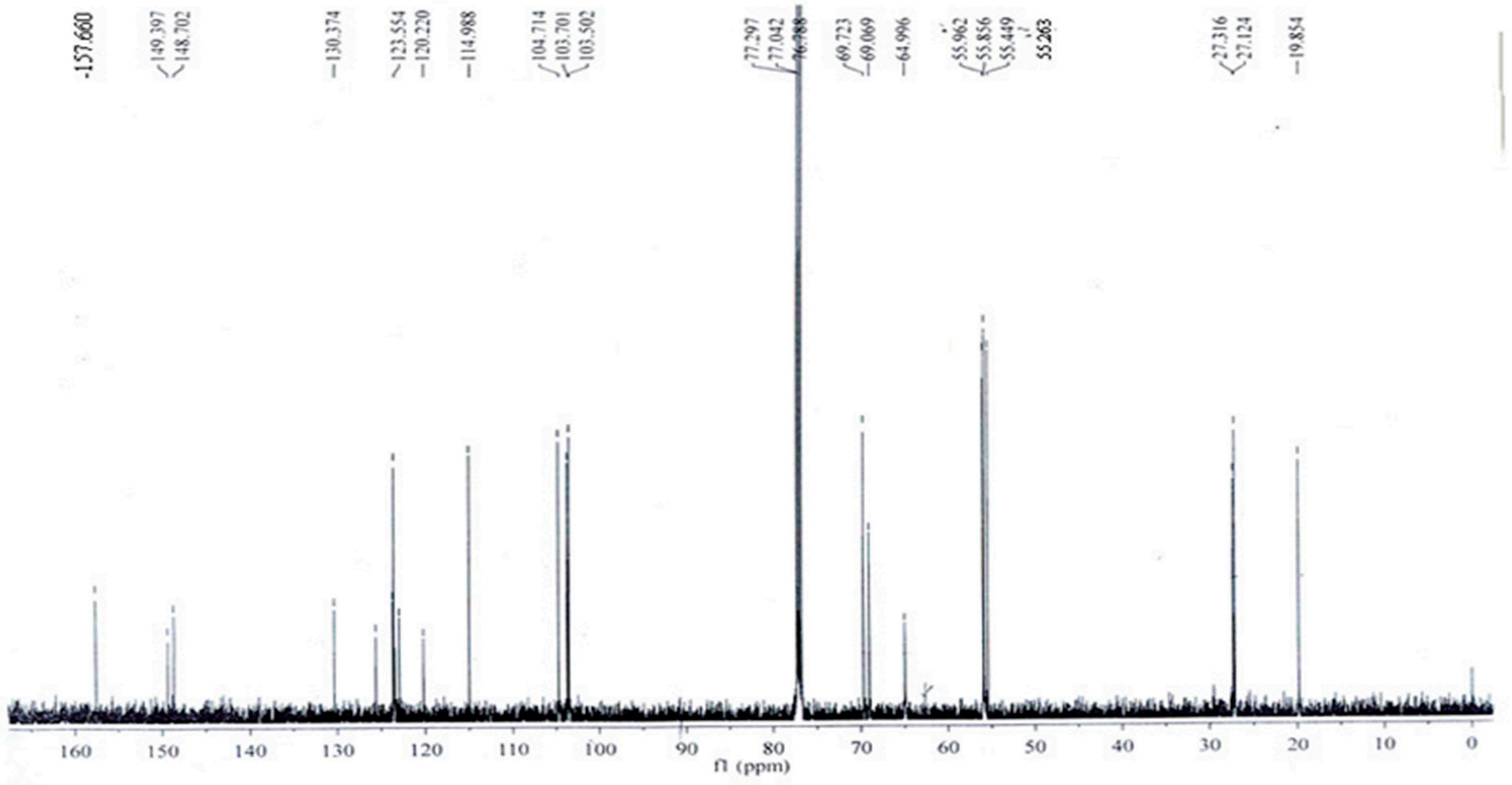

Figure S6. ^{1}H-NMR spectra of tylophorine.
